# Editorial: Psychotic experiences, social cognition and pragmatic communication in the psychosis continuum

**DOI:** 10.3389/fpsyg.2023.1127477

**Published:** 2023-02-08

**Authors:** Alberto Parola, Marta Bosia, Guillermo Soto, Ricardo Garcia

**Affiliations:** ^1^Department of Linguistics, Cognitive Science and Semiotics, Aarhus University, Aarhus, Denmark; ^2^The Interacting Minds Center - Institute of Culture and Society, Aarhus University, Aarhus, Denmark; ^3^Department of Psychology, University of Turin, Turin, Italy; ^4^School of Medicine, Vita-Salute San Raffaele University, Milan, Italy; ^5^Department of Clinical Neurosciences, IRCCS San Raffaele Scientific Institute, Milan, Italy; ^6^Department of Linguistics, Center of Cognitive Studies, University of Chile, Santiago, Chile

**Keywords:** pragmatics, psychosis, schizophrenia, psychotic experience, social cognition, communication disorders, psychosis continuum

## Introduction

**Figure 1:**
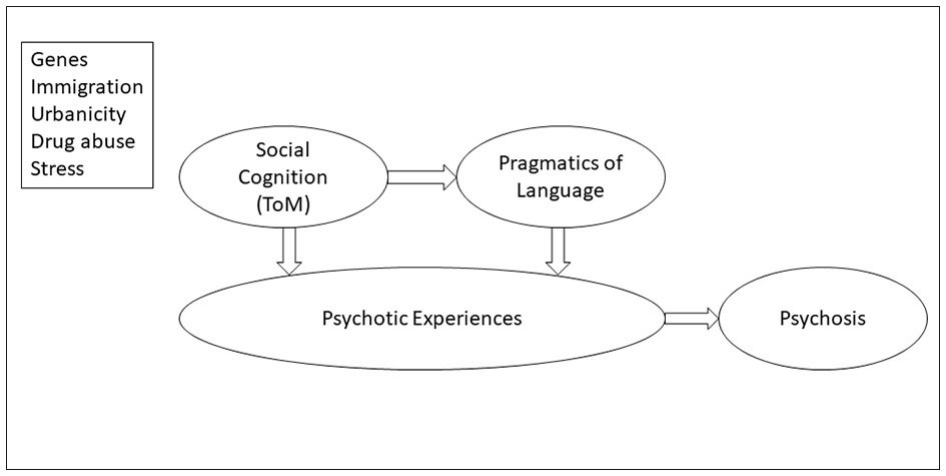
Psychotic experiences, social cognition, and pragmatics of language in the psychosis continuum. Social cognition (primarily, theory of mind) and pragmatics of language are associated with psychotic experiences and the transition to psychosis (Clemmensen et al., 2016; Sullivan et al., 2016). Social cognition determines, at least in part, linguistic pragmatics (Sullivan et al., 2016). The top panel shows risk factors for psychotic experiences and psychosis (schizophrenia): genes, immigration, urbanicity, drug abuse and stress.

Psychotic experiences (PE), such as perceptual abnormalities and unusual beliefs, are widespread in the general population, but they often remain below the psychosis diagnostic threshold (Kelleher and Cannon, 2011; Linscott and Van Os, 2013). However, PE may represent a first point along a psychosis continuum, and identifying them in childhood and adolescence is crucial because of their association with an increased risk of developing psychosis, and with other psychosis-related features including social and cognitive disorders (Gregersen et al., 2022). In particular, deficits of social cognition and pragmatic communication seem to have a central role in the emergence and maintenance of PE in non-clinical populations, and can crucially contribute to transition to psychosis (e.g., Agostoni et al., 2021; Parola et al., 2021, see Figure 1).

In this Research Topic (RT), we aimed to gather new evidence and critical reappraisal from researchers addressing the relationship between PE and deficits of social cognition and pragmatic communication.

We focused on the nature of the interacting factors that determine the persistence of PE and the risk of transition to psychosis, and the relation between social cognition/pragmatic communication and other relevant cognitive and social domains in the context of PE.

## The Research Topic

The RT is composed of 11 articles covering original research to reviews, conceptual analysis, and theoretical and perspectives articles from 74 researchers working in 12 different countries. More in detail, the main themes that emerged and were addressed are:

1) Psychosocial and cognitive factors that determine the development or persistence of psychotic experience in the psychosis spectrum and in subclinical populations.

- Krijnen et al. investigated the association between closeness of social contact, positive and negative affect and symptoms, and empathy in a sample of individuals with psychosis and healthy controls. The authors found that social contact, and especially contact with a close other, is beneficial for positive affect in the total sample and for positive symptoms in individuals with psychosis.
- Beals et al. aimed to characterize the relationship between paranoia, empathy and emotion recognition in a non-clinical sample of adults. They found that only a specific facet of cognitive empathy, i.e., imaginative perspective-taking, is related to paranoia in the general population; however, the association between empathy and paranoia did not appear to depend on emotion recognition. The author concluded that deficits in empathy and emotion recognition observed in schizophrenia may not be similarly detectable in subclinical populations.
- Csulak et al. present a meta-analysis and systematic review involving implicit mentalization in schizophrenia. Interestingly, the meta-analysis showed slower reaction time and low accuracy during implicit mentalization in schizophrenia. The systematic review also revealed different brain activation patterns and visual sensory motor alteration. Implicit mentalization alteration seems to play a role in schizophrenia, although not to the same extent as explicit mentalization.
- Chapellier et al. using the Mini Profile of Non-verbal Sensitivity, revealed that non-verbal social perception is impaired in patients with schizophrenia compared to controls, and that it is also related to the severity of psychopathology and functional disruption. These results underline the need for novel therapeutic approaches to alleviate non-verbal social perception deficits.
- Giugliano et al. using the Metacognition Self-Assessment Scale (MCAS) in a non-clinical population, revealed that metacognitive skills play a protective role in the occurrence of psychotic-like experiences in non-clinical individuals.
- Cavieres and López-Silva provided a precise and unitary definition of social perception, suggesting that it refers to low-level pre-reflective processes underlying the awareness of interpersonal interactions with and between others. The authors suggest that a better identification and assessment of this domain may be beneficial for developing new psychosocial rehabilitation programs.
- Bob et al. found, in a sample of drug naive women in their first psychotic episode, a relationship between epileptic-like symptoms and measures of chronic stress. Indeed, recent evidence indicates that early stressful and traumatic experiences may impact on the brain and increase the risk for psychosis, leading to neural processes similar to epilepsy which may occur in patients with mental disorders including schizophrenia. The present study provides useful information for diagnostic consideration of anticonvulsant therapy, also supporting its use in patients who do not respond to usual psychotropic medication.

2) New rehabilitative treatments aimed at recovering social and pragmatic deficits observed in the psychosis continuum.

- Lado-Codesido et al. conducted a randomized, single-blind, multicenter clinical trial to test the effectiveness of the “Voices 2” training program to improve emotion recognition through prosody in adults with schizophrenia. The authors found an improvement in recognition of emotional prosody in the patients which undergone the treatment, but not in control group.

3) New assessment tools aimed at detecting psychosocial factors that determine the development or persistence of psychotic experience.

- Kim et al. validated a Korean version of The Anticipatory and Consummatory Interpersonal Pleasure Scale (ACIPS), a psychometric scale used indirectly to measure social anhedonia. The results showed that ACIPS revealed to be a useful psychometric instrument in non-help-seeking populations.

4) Neurobiological abnormalities associated with neuropsychiatric conditions and their symptoms.

- Sun et al., found that adult patients with early and late onset recurrent depression, compared to healthy controls, have abnormal neuronal fMRI activity in some brain regions, with differences closely related to the default mode network, and the salience network, and that patients of each age group exhibit regional homogeneity abnormalities relative to matched controls. The prevalence of depressive disorders in schizophrenia is reported to be very high (~40%), and these findings are therefore of great importance and relevance, even when taking into account the neurobiological abnormalities associated with schizophrenia spectrum disorder.
- Morese et al. found that patients with SCZ, compared to healthy controls, showed a higher fMRI neural activation of the left-middle temporal gyrus (L-MTG) while performing a communicative pragmatic task; the involvement of the L-MTG was related to the increasing inferential effort required in correctly understanding the speaker's communicative intentions, and the higher integrative semantic processes involved in sentence processing.

## Conclusions

This RT has contributed to identify which factors may contribute to the occurrence and persistence of PE in both the general population and the psychosis spectrum, and which aspects may instead play a protective role and reduce the risk of transition to psychosis.

The RT has explored the relationship between social cognition, pragmatic communication, and other relevant cognitive and social domains associated with PE and has shown, for example, that implicit mentalization and non-verbal social perception are two key skills that selectively distinguish patients with schizophrenia from controls.

Crucially, the RT has also revealed new ways to assess, conceptualize, and rehabilitate the psychosocial, cognitive, and clinical aspects involved in the development and persistence of PE. Indeed, new validated assessment tools—the ACIPS scale–, new conceptual views and new intervention treatments—the Voices 2 training program—were presented in the RT.

In summary, the RT has helped to uncover some of the complex interactions between multiple factors underlying the onset of PE and the transition to psychosis, and provided support to the idea of a continuum between the general population and the psychosis spectrum. Finally, the RT has also shown what is currently lacking and where future efforts might be directed: longitudinal design are essential to track over time the relationships between PE and deficits in social cognition and pragmatic communication and the onset of psychosis. We also need prospective studies that can track the development of PE since infancy and childhood and identify early signs of PE and psychosis and their association with clinical and psychosocial factors (Fried et al., 2022; Thorup et al., 2022).

## Author contributions

All authors listed have made a substantial, direct, and intellectual contribution to the work and approved it for publication.
